# Editorial: Fostering Creative Organizations: Antecedents, Processes, and Consequences of Individual and Team Creativity

**DOI:** 10.3389/fpsyg.2019.02840

**Published:** 2019-12-17

**Authors:** Sujin Lee, Mary C. Kern, Sukanlaya Sawang

**Affiliations:** ^1^College of Business, Korea Advanced Institute of Science and Technology (KAIST), Daejeon, South Korea; ^2^Zicklin School of Business, Baruch College (The City University of New York), New York, NY, United States; ^3^Coventry Business School, Coventry University, Coventry, United Kingdom

**Keywords:** individual creativity, team creativity, dyadic creativity, workplace creativity, organizational creativity

Creativity, defined as the ability or process to generate novel and useful ideas, is the key engine of organizational innovation. It is a critical differentiator of mind from machine, a core driver of our uniqueness in an increasingly automated world. An important question, then, is how best to improve people's creativity, to enhance our collective innovativeness, enjoyment, and global living standards. Despite ample attention—including theory- and application-focused research—to this question, there remains much to learn about creativity.

Specifically, while prior work has focused on creativity's dispositional and situational predictors, we need to know more about other antecedents that boost or hamper individual, dyadic, and team creativity in organizational settings. This Research Topic's goal is to provide a forum to discuss new ideas and recent theoretical developments in creativity from multiple levels, with practical implications for fostering creative organizations. Eight papers were published in our Research Topic: seven empirical studies and one review paper.

Two of the papers elucidate motivational antecedents of individual creativity. Kung and Scholer examines how goals are structured—as opposed to their content—and their impact on individuals' ability to combine ostensibly unrelated ideas into something novel and meaningful. Participants were asked to organize their focal goal (related to academic success) relative to other idiosyncratic goals in one of three structures (network, hierarchical, sequential). Results revealed that a network mindset produced more convergent creativity. This work highlights multiple non-obvious connections across and between goals, and suggests that how people (and organizations) structure their goals has downstream implications for individual creativity.

Unlike intrinsic motivators such as goals, extrinsic motivators have been shown to have harmful or minimal effects on individual creativity. Fischer et al. thus addressed a gap involving the creativity-motivating role extrinsic factors can play—specifically, the synergistic extrinsic motivators that organizations can use to foster creativity and innovation in their intrinsically motivated knowledge workers. The authors found that their extrinsic motivator (relational rewards) moderated significantly the link between intrinsic motivation and creativity/innovation performance: the higher the perceived probability of receiving relational rewards and the higher the intrinsic motivation, the greater the positive effect.

In contrast to individual creativity, how to promote dyadic, interpersonal creativity has received little attention, despite the ubiquity of human collaboration today. Kung and Chao examines how contrasting emotions between partners influence creative solutions in dyadic negotiation. Experimental data from 105 dyads showed that mixed-emotion (happy/angry) dyads generated more joint creative performance than same-emotion (happy/happy, angry/angry) dyads. This work extends the creativity-boosting effect of mixed emotion from the individual to the dyadic level. Organizations may benefit from strategic use of employee emotion, for example, by matching workers with contrasting emotional traits for a creativity-requiring project, or developing emotion-regulation training for collaborating colleagues.

Coordination of behavior among dyad members also influences creativity. Gaggioli et al. studies the carryover effect of sensorimotor synchronization on a subsequent creative dyadic task. Results show that unacquainted male dyads were more creative after a joint-action coordination task; female dyads and mixed (male-female) dyads were not. This suggests organizations should use a coordinated sensorimotor activity to promote creativity for male dyads, but not for female/mixed dyads, highlighting the important, yet largely unknown role of behavioral synchronization in dyadic creativity.

One of the most common, consequential forms of dyadic creativity is follower creativity, as influenced by leadership. Wang et al. examines the cross-level psychological mechanisms explaining the impact of humble leadership on follower creativity. Analyzing time-lagged data of 328 team members nested within 1–6 teams, the authors found that humble leadership promoted follower creativity only when both psychological safety and knowledge sharing are present. This study untangles complex cross-level relationships to suggest that organizations should educate and train managers and leaders on the important role of humility in facilitating follower creativity.

Bastian et al. untangles the effect of shared adversity on team creativity. Experimental data from interactive teams showed that sharing a painful (vs. non-painful) experience increased supportive interaction among teammates, subsequently promoting newly formed teams' creativity. This research complements prior research highlighting beneficial effects on group process of sharing positive experience within teams. It appears that shared negative experience also promotes team creativity, something important for managers to understand.

Another paper studies predictors for *organizational* innovation. Li and Yu explores the structure and antecedents of the path to innovation. Results indicate that intellectual capital has a direct, positive effect on technological innovation, but no direct effect on business model innovation. Organizational character mediates the beneficial effect of intellectual capital on both technological innovation and business model innovation. By considering intellectual capital and organizational character common antecedents of innovation, and breaking innovation into technological innovation and business model innovation, the study establishes a new, important analysis framework for dual innovation, enriching innovation theory.

Finally, the review paper Paulus et al. synthesizes theoretical and empirical work on organizational innovation and team processes, to untangle the conditions that facilitate collaborative organizational innovation. By integrating literature from different levels of analyses, this work suggests a pragmatic potential application for enhancing organizational innovation.

In sum, we wish to gain more comprehensive understanding of individual/dyadic/team creativity, to foster more creative interactions and organizations. All papers in our Research Topic address antecedents of creativity. Still, unknown predictors of creativity remain, and we need additional research on other, novel antecedents of dyadic and team creativity, as well as on its process and consequences. We should also learn more about how to make creative ideas work—from idea-generation to implementation.

In conclusion, this Research Topic highlights the central question of how best to foster creative organizations at the individual, interpersonal, and team levels, a critical priority for innovation-seeking organizations worldwide.

## Author Contributions

All authors listed have made a substantial, direct and intellectual contribution to the work, and approved it for publication.

### Conflict of Interest

The authors declare that the research was conducted in the absence of any commercial or financial relationships that could be construed as a potential conflict of interest.

